# Differences in the Cellular Immune Response during and after Treatment of Sudanese Patients with Post-kala-azar Dermal Leishmaniasis, and Possible Implications for Outcome

**DOI:** 10.1007/s44197-024-00270-0

**Published:** 2024-07-15

**Authors:** Ana Torres, Brima Musa Younis, Mohammed Alamin, Samuel Tesema, Lorena Bernardo, Jose Carlos Solana, Javier Moreno, Alaa-aldeen Mustafa, Fabiana Alves, Ahmed Mudawi Musa, Eugenia Carrillo

**Affiliations:** 1 WHO Collaborating Centre for Leishmaniasis, Spanish National Center for Microbiology, Instituto de Salud Carlos III (ISCIII), Majadahonda (Madrid), Spain; 2 CIBER de Enfermedades Infecciosas, Instituto de Salud Carlos III, Madrid, Spain; 3 Department of Clinical Pathology & Immunology, Institute for Endemic Diseases, University of Khartoum, Khartoum, Sudan; 4 Drugs for Neglected Diseases Initiative, Nairobi, Kenya; 5 Drugs for Neglected Diseases Initiative, Geneva, Switzerland

**Keywords:** PKDL, Leishmaniasis, Whole blood assay, Cytokines, Outcome, Treatment

## Abstract

**Background:**

The host cellular immune response associated with two treatments for post-kala-azar dermal leishmaniasis (PKDL) - paromomycin plus miltefosine (Arm 1), and liposomal amphotericin B plus miltefosine (Arm 2) - was examined in Sudanese patients before treatment (D0), at the end of treatment (D42), and during the post-treatment period (D180).

**Methods:**

Whole blood samples were stimulated with soluble *Leishmania* antigen for 24 h (whole blood assay [WBA]) and the concentrations of Th1/Th2/Th17-associated cytokines, IP-10, PDL-1 and granzyme B were determined.

**Results:**

The Arm 1 treatment (98.2% cure rate) induced a Th1/Th2/Th17 response, while the Arm 2 treatment (80% cure rate) induced a Th1/Th2 response. Five Arm 2 patients relapsed and showed lower IFN-γ, TNF and IL-1β concentrations at D0 than non-relapsers in this Arm. In patients with low-IFN-γ-production at D0, Arm 1 treatment led to a better host immune response and clinical outcome than Arm 2 treatment.

**Conclusions:**

A Th1/Th2/Th17 response was associated with a higher cure rate. Patients with low IFN-γ, TNF and IL-1β before treatment are more likely to relapse if they undergo Arm 2-type treatment. Determining IFN-γ, TNF and IL-10 levels prior to treatment could help predict patients at higher risk of relapse/recovery from PKDL.

**Trial Registration:**

ClinicalTrials.gov NCT03399955, Registered 17 January 2018, https://clinicaltrials.gov/study/ NCT03399955.

**Graphical Abstract:**

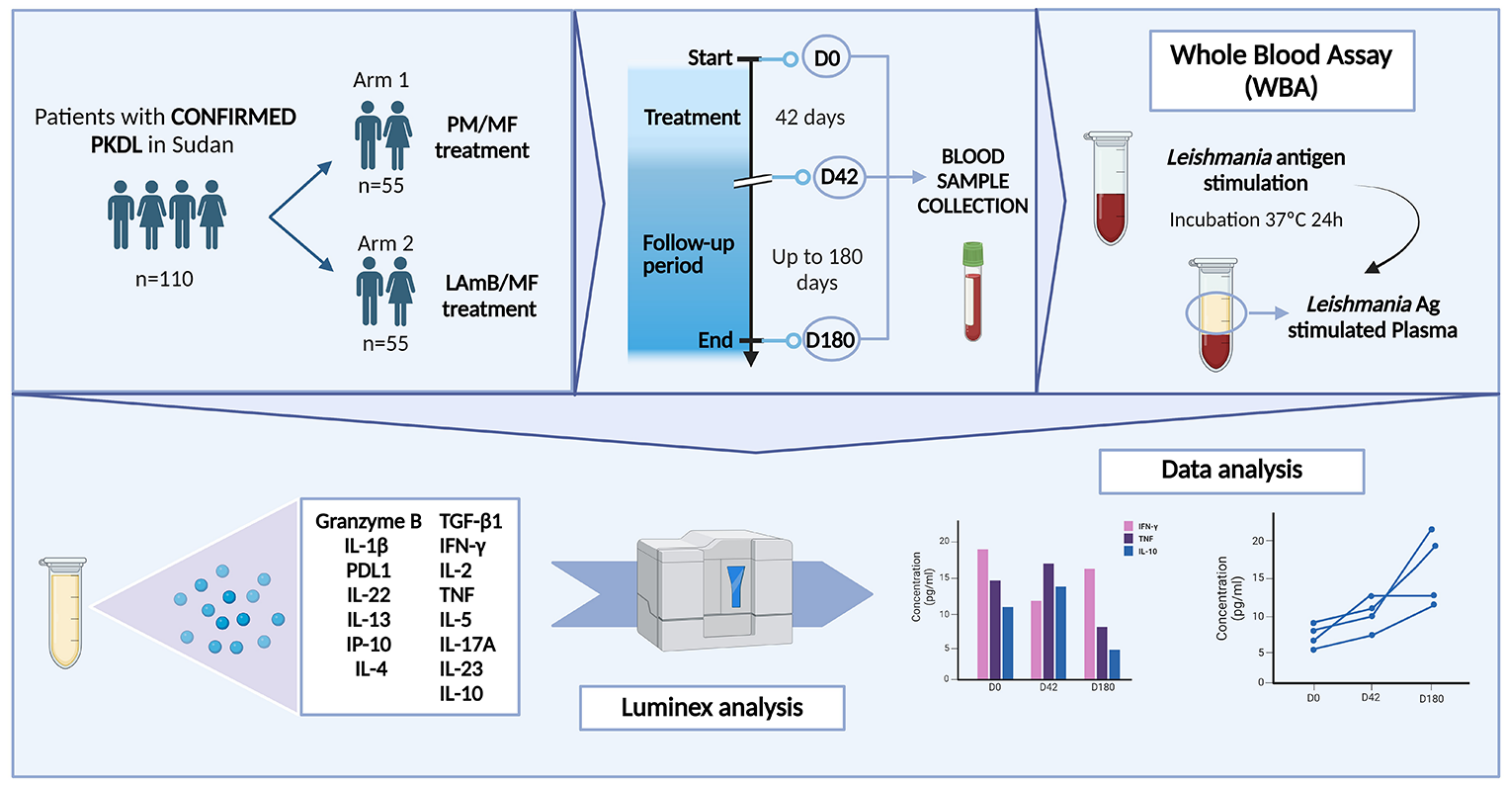

**Supplementary Information:**

The online version contains supplementary material available at 10.1007/s44197-024-00270-0.

## Highlights

No blood biomarker can currently predict treatment outcome for patients with PKDL.

The cellular response during treatment is key to overcoming leishmaniasis.

Patients who relapsed showed low SLA-induced production of IFN-γ, TNF and IL-1β.

Treatment effectiveness was related to higher IFN-γ production and absence of IL-10.

PKDL treatment can be monitored and relapse predicted by the whole blood assay.

**Supplementary Information:**

The online version contains supplementary material available at 10.1007/s44197-024-00270-0.

## Introduction

Post-kala-azar dermal leishmaniasis (PKDL) may appear after treatment for visceral leishmaniasis (VL) caused by *Leishmania donovani* [1, 2]. The Indian subcontinent, especially India, Nepal and Bangladesh, has a high PKDL burden, while in East Africa Sudan, followed by Ethiopia, record the highest incidence. Indeed, historically, some 50–60% of Sudanese patients with VL developed PKDL following sodium stibogluconate (SSG) monotherapy [1–5]. A recent study reports PKDL to be less common in VL patients when treated with SSG plus paromomycin (20.9%), or with miltefosine (MF) plus paromomycin (PM) (4.4%) combination therapies. In Sudan, PKDL is first manifested as a skin rash with nodular or papular lesions, usually on the face (Grade 1). It may later spread to other areas (Grade 3 refers to the whole body being affected). The lesions may resolve spontaneously within 6 to 12 months, but in some patients they can persist for decades. While the age distribution for PKDL in Sudan is identical to that of VL, young children (< 6 years) may develop particularly severe and generalized skin lesions [2, 6].

Since PKDL skin lesions harbour leishmanial parasites, treatment is essential if the spread of leishmaniasis is to be controlled. However, given the toxicity of current PKDL treatments, the only patients treated are those with severe disease or chronic lesions that persist after 6 months, those with concomitant anterior uveitis, and young children with oral lesions that interfere with feeding [3, 4, 7, 8]. In addition, PKDL lesions are usually not itchy or painful, and the condition is not life-threatening, therefore patients in Sudan may not seek medical care [9] and thus remain a reservoir of infection. The most commonly prescribed treatment in Sudan is SSG (20 mg/kg/day) for 40–60 days or longer (possibly up to 90–120 days for severe and persistent lesions). Unfortunately, this requires painful, daily, intramuscular or intravenous injections and thus the need to remain close to medical facilities over a lengthy period. A further problem lies in the toxicity of SSG, which can be life-threatening, especially for long treatment duration [10]. New drugs and more effective short drug combinations are urgently needed, but clinical studies in this line have been scant, and those that have been performed have been limited to small cohorts [11].

Since leishmaniasis is an intracellular infection, the cellular immune response plays a crucial role in disease progression and cure. The few studies that have examined the systemic immune response in Sudanese patients with PKDL [12–14] have revealed that, when lesions are present, peripheral blood mononuclear cells (PBMCs) proliferate and produce IFN-γ and IL-10 in response to leishmanial antigens, indicating a mixed immune response responsible for both skin inflammation and the persistence of *Leishmania* [12, 13]. However, little is known about the specific cellular immune response associated with the treatment and cure of PKDL. The present work reports the changes in the cellular immune response profiles over treatment and follow-up of patients participating in a phase II clinical trial to assess two new combination treatments for patients with PKDL in Sudan, and correlation of immune response with treatment outcomes.

## Methods

### Study Design

This clinical trial was conducted between 2018 and 2021 at Professor El-Hassan Centre for Tropical Medicine (Doka, Gadarif State, Sudan), a reference centre for the treatment of VL and PKDL. All the participating patients (total *N* = 110; 69 male, 41 female; age 6–60 years; median age 9 years [IQR 7–10]; all from Gadarif State) had PKDL initially suspected by clinical presentation and confirmed by the detection of parasites, either by microscopic examination of a skin smear, or by PCR. All had stable or progressive disease for at least 6 months, or Grade 3 PKDL disease. These patients were randomly assigned to receive either intramuscular paromomycin (20 mg/kg/d) for 14 days plus oral miltefosine (allometric dose) for 42 days (PM/MF; Arm 1; *n* = 55), or liposomal amphotericin B (LAmB [AmBisome^®^], intravenous, 20 mg/kg total dose) over 7 days plus oral miltefosine (allometric dose) for 28 days (LAmB/MF; Arm 2; *n* = 55). Full details regarding the selection and randomisation procedures, plus information on treatment efficacy and safety, are available at Younis et al., 2023 [15].

### Clinical Assessment

All patients underwent clinical assessment before treatment began (D0), at day 42 after the start of treatment (D42), at day 180 (D180), and at 12 months, by expert clinicians from Doka. Disease progress was recorded using the PKDL grading scale, which assesses disease severity by lesion distribution and density (Grades 1–3) [15]. Additionally, haematological and biochemical parameters were evaluated, including albumin, creatinine, potassium, SGOT/AST, SGPT/ALT, total bilirubin, hemoglobin, platelets, RBC, WBC, and percentages of basophils, eosinophils, hematocrit, lymphocytes, monocytes, and neutrophils.

### Stimulation of Whole Blood with ***Leishmania*** Soluble Antigen

Blood samples for immunological studies (3 ml in heparinized tubes) were collected at D0, D42 and D180. No blood sample collection was scheduled at 12 months, only clinical examination was performed at this time. Following a previously described procedure [16, 17], one 500 μl aliquot of whole blood from each sample was stimulated with *Leishmania* soluble antigen (SLA, 10 μg/ml), another was stimulated with phytohemagglutinin (PHA, 10 μg/ml) as a positive control, and another left unstimulated as a negative control. All samples were then incubated at 37 °C for 24 h. After incubation, the supernatants were collected and stored at -80 °C until analysis.

### Cytokine/Other Analyte Determinations

To examine the specific cellular immune response associated with the two treatments, the above supernatants were examined for 10 cytokines and two other analytes (IFN-γ, IL-1β, IL-2, IL-4, IL-5, IL-13, IL-17A, IP-10, PD-L1, TNF, IL-10 and granzyme B) using the magnetic Human XL Cytokine Luminex^®^ Performance Base Kit from R&D Systems (Minneapolis, MN, USA) (cat# LUXLM000). IL-22 and IL-23 were determined using the Human IL-22 High Sensitivity Magnetic Luminex^®^ Performance Assay Kit, and the Human IL-23 High Sensitivity Magnetic Luminex^®^ Performance Assay Kit, respectively, also from R&D (cat# LBHS5782 and LBHS1716). TGF-β1 was determined using the TGF-beta 1 Magnetic Luminex^®^ Performance Assay Kit from the same supplier (cat# LTGM100). All kits were used following the manufacturer’s instructions. Data were acquired using the Bio-Plex^®^ 200 System (Bio-Rad, CA, USA) with automatic clustering, and analysed using Bio-Plex Manager Software (Bio-Rad). Results for each cytokine/other analyte concentration (expressed in pg/ml) were determined and expressed either as the difference between SLA-stimulated and control plasma concentrations, or as individual control and SLA-stimulated concentrations directly, as indicated.

### Statistical Analysis

The statistical analysis was performed on the modified intention-to-treat (mITT, all randomized patients receiving at least one dose of treatment; in case of erroneous treatment allocation, the actual treatment received was to be used in the analysis). Normality was examined using the Shapiro-Wilk test. The differences within arms, between the two arms, and between groups (e.g., when comparing differences between patients with different grades of disease) were analysed using the Mann-Whitney U test (two-tailed). Differences between patients who relapsed and those who did not were examined using the same test (one-tailed). All statistical analyses were performed using GraphPad Prism v.9.0 software (GraphPad Software, La Jolla, CA, USA).

## Results

A total of 110 patients were included in the mITT analysis, with samples available for all patients at D0, 104 patients at D42, and 72 patients at D180. The most common type of lesion seen was maculopapular (77.3% of all patients; Arm 1: 41/55 [74.5%] and Arm 2: 44/55 [80.0%]). The majority of patients had grade 1 PKDL lesions in both distribution (Arm 1: 43/55 [78.2%], Arm 2: 41/55 [74.5%]) and density (Arm 1: 35/55 [63.6%], Arm 2: 36/55 [65.5%]). The remaining patients had grade 2 and 3 lesions, with 21.8% (12/55) distribution and 36.4% (20/55) density in Arm 1, and 25.5% (14/55) distribution and 34.5% (19/55) density in Arm 2 [15].

All patients included in the study had a prior diagnosis of VL and received different treatments, either sodium stibogluconate (SSG) alone or in combination with paromomycin (PM). The majority of patients received SSG & PM (Arm 1: 32/55 [58.2%] and Arm 2: 34/55 [61.8%]), the rest received SSG alone (Arm 1: 22/55 [40.0%] and Arm 2: 21/55 [38.2%]). Notably, only one patient in Arm 1 did not have a documented record of the VL treatment received. Definitive cure at 12 months was achieved in 98.2% of the PM/MF-treated patients (Arm 1) and 80% of LAmB/MF-treated patients (Arm 2) in the mITT analysis, while all patients (100%) achieved cure in Arm 1 and 86% in Arm 2 in the set of completers analysis (excluding patients with missing 12-months efficacy outcome). Seven Arm 2 patients experienced treatment failure and required rescue treatment. Among those, six had Grade 2 and 3 lesions at D0, and five of these six relapsed by 12 months of follow-up [15].

### Cytokine/Other Analyte Response before and after Treatment (all Patients as a Whole)

**Fig. 1:**
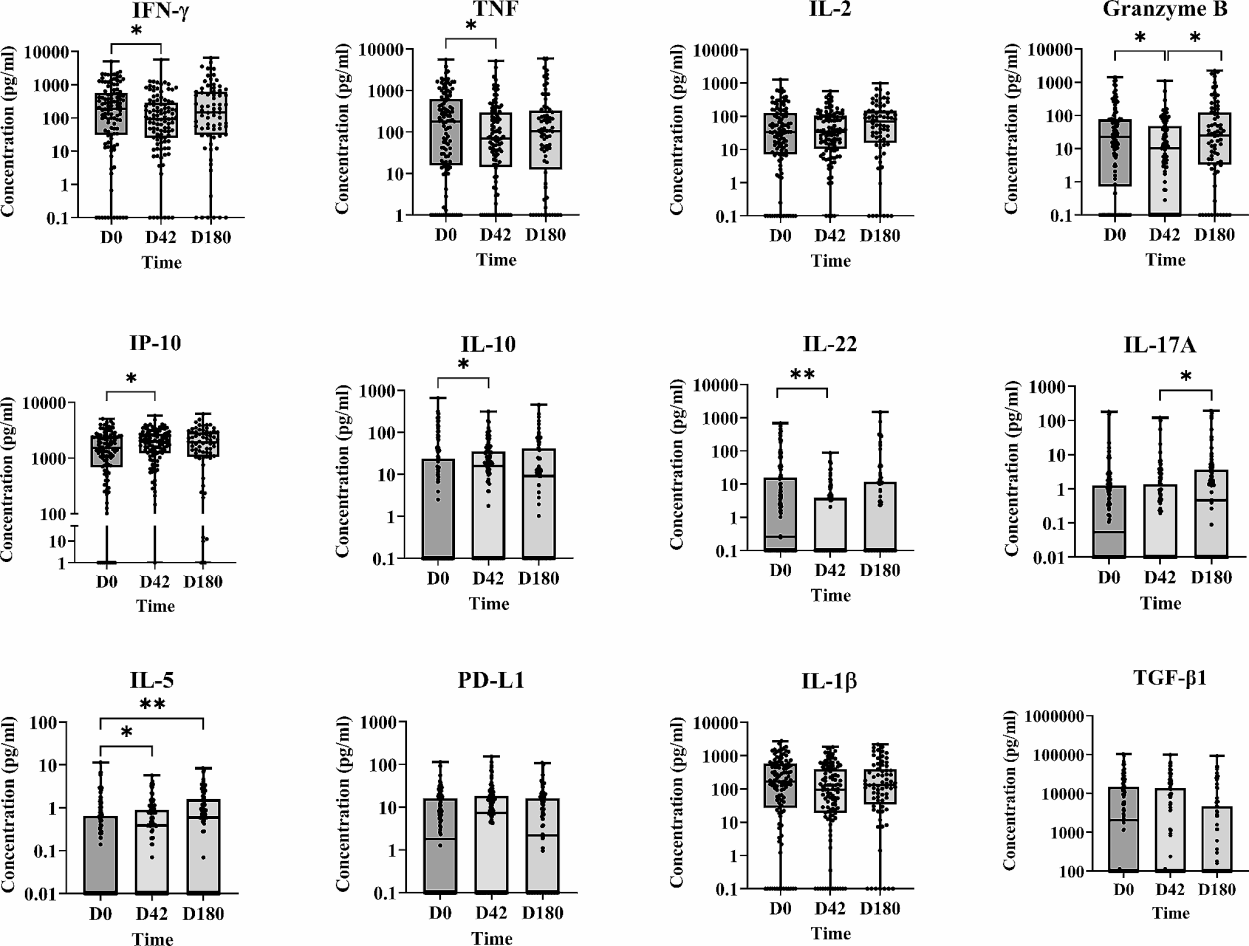
Cytokine, PD-L1 and granzyme B in plasma from all 110 patients in the mITT set, in response to SLA stimulation (as determined by WBA). IFN-γ, TNF, IL-2, granzyme B, IP-10, IL-10, IL-22, IL-17A, IL-5, PD-L1, IL-1β and TGF-β1 concentrations (pg/ml) were measured at D0 (110 patients), D42 (104 patients) and D180 (72 patients) and the data reported is the difference between SLA and control. Statistical differences (Mann-Whitney test): *p<0.05, **p<0.01

Prior to treatment (D0), stimulation with SLA revealed that most of the total 110 patients produced proinflammatory cytokines, mainly IFN-γ and TNF, IP-10 and granzyme B [median (IQR) = 187.80 (0-5038); 175.60 (0-5573); 1506 (0-5041); 22.37 (0-1409), respectively] (Fig. 1). In contrast, initial levels of anti-inflammatory cytokines such as IL-5, IL-13 or IL-10 were close to zero. The notable exception was TGF-β1, for which high level production was recorded [median (IQR) = 2011 (0-103,847)] (Fig. 1, Table S1). At the end of treatment (D42), the above proinflammatory Th1 profile was slightly reduced except for IP-10. While the median concentrations of IFN-γ, TNF and granzyme B decreased significantly [median (IQR; *p value*) = 94.13 (0-5639; <0.05); 68.15 (0-5093; <0.05); 10.14 (0-1088; <0.05), respectively], the production of IP-10, IL-5 and IL-10 increased [median (IQR; *p value*) = 1941 (0-5809; <0.05); 0.39 (0-5.71; <0.05); 15.71 (0-311.50; <0.05), respectively], although IL-5 and IL-10 were expressed at very low levels (Table S1). In addition, production of the Th17-related cytokine IL-22 was reduced at D42 [median (IQR; *p value*) = 0.0 (0-86.73; *p =* 0.0016)]. Similarly, the median concentration of TGF-β1 was close to zero [median (IQR) = 0 (0–98,858)]. At follow-up (D180), tendency towards a reactivation of the cellular immune response was noted, with higher concentrations of granzyme B and IL-17A compared to D42 [median (IQR; *p value*) = 24.47 (0-2210; <0.05); 0.46 (0-192; <0.05), respectively]. An increase in IL-5 concentrations over those measured at D0 was also noted. At first glance, an apparent recovery in IFN-γ, TNF and IL-1β production was noted at D180 compared to D42, suggesting a Th1/Th2/Th17 profile, but these changes were not significant. Importantly, the median TGF-β1 concentration detected at D0 remained low at D180. In summary, despite all the changes in the cellular immune response, at D180 the overall patients showed a similar cytokine profile as the one observed before treatment, except for increased IL-5 production (albeit at a very low level) (Table S1).

No differences in cytokine production were seen between patients with Grade 1 and Grade 2/3 lesions at D0 or any other time. Thus, lesion severity was not directly associated with a specific cytokine profile (Suppl. Figure 1).

No significant differences were observed in median cytokine concentrations between the two Arms at any time. The following lines describe the changes observed within each arm during the treatment and follow-up.

### Effectiveness of PM/MF Treatment: The Elicited Th1/Th2/Th17 Response Indicates Likely Cure

**Fig. 2:**
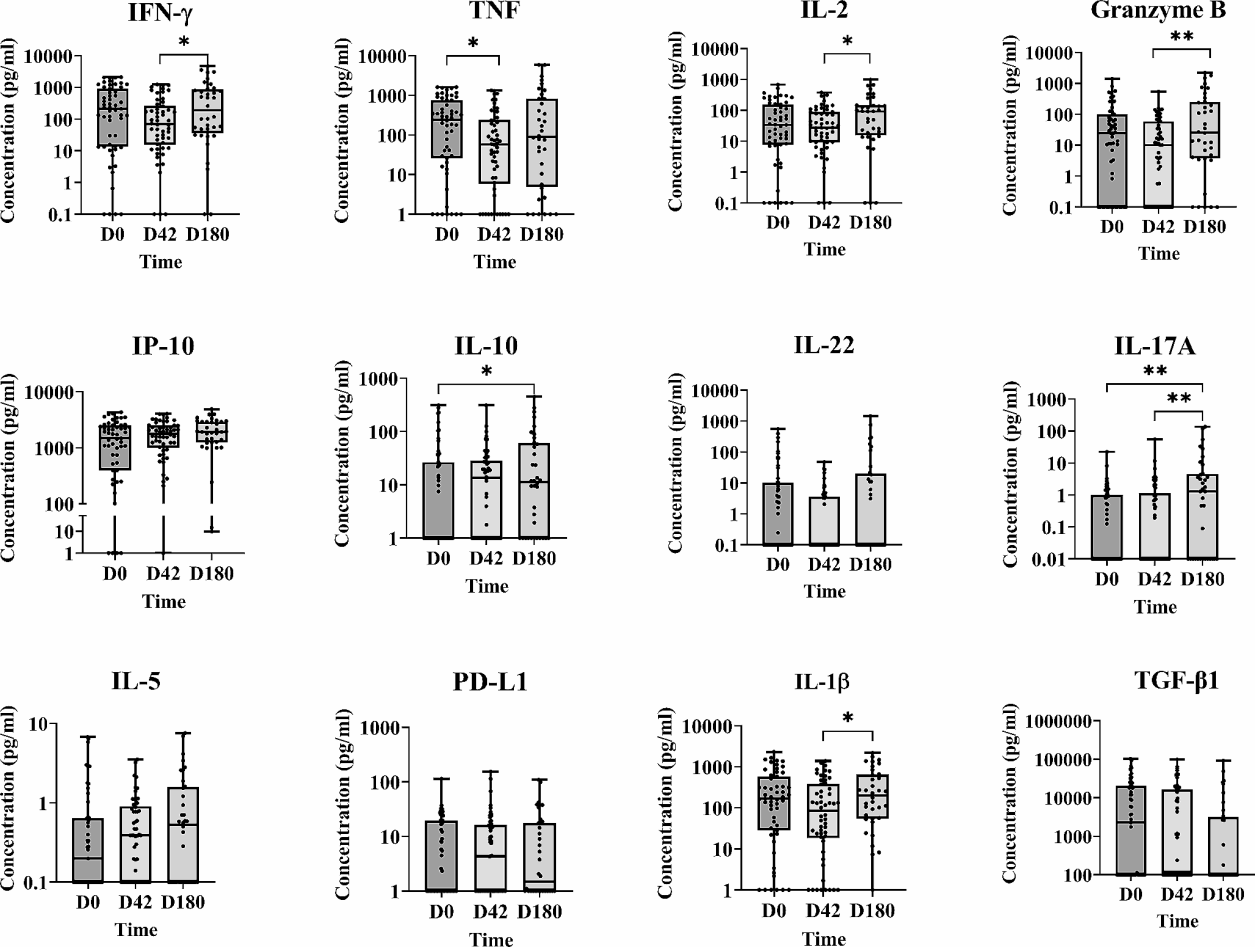
Cytokine, PD-L1 and granzyme B concentrations in PM/MF-treated (Arm 1) patients (as determined by WBA) in the mITT set. IFN-γ, TNF, IL-2, granzyme B, IP-10, IL-10, IL-22, IL-17A, IL-5, PD-L1, IL-1β and TGF-β1 concentrations (pg/ml) were measured at D0 (55 patients), D42 (54 patients), and D180 (36 patients). Statistical differences (Mann-Whitney test): *p<0.05, **p<0.01

At D0 the patients treated with PM/MF showed a cellular response similar to the one observed when taking all patients together (Fig. 2). For these Arm 1 patients, no significant changes were observed between D0 and D42 in terms of median concentrations of Th1 cytokines, except for a significant reduction in TNF [median (IQR; *p value*) D0 = 244.2 (0-1612); D42 = 57.63 (0-1342; *p* = 0.0141)]. At D180, a significant increase was seen in the concentrations of IFN-γ, IL-2, granzyme B, IL-1β and IL-17A compared to D42 [(median (IQR; *p value*) D42 vs. D180 = 69.85 (0-1244; <0.05) vs. 190.90 (0-4726); 27.34 (0-377.7; <0.05) vs. 91.67 (0-989.1); 10.14 (0-540.1; <0.01) vs. 25.93 (0-2210); 84.48 (0-1391; <0.05) vs. 204 (0-2193); 0 (0-55.20; <0.01) vs. 1.29 (0-137), respectively)]. The cytokine profile at D0 was also similar to the one at D180, except for IL-10 and IL-17A which were already slightly increased at D42 (indicating a Th1/Th2/Th17 response). TGF-β1 decreased gradually from D0 [median (IQR) = 2271 (0-103,847) at day 0 vs. 119.40 (0–98,858) at day 42], becoming undetectable by D180 [(median (IQR; *p value*) = 0 (0–92,407; *p* = 0.064)] (Table S1).

All patients treated with PM/MF who completed the study were declared cured at 12 months, irrespective of the initial severity of their disease (one patient was lost to follow-up). No relapse was observed during follow-up to 12 months.

### Effectiveness of LAmB/MF Treatment: the Elicited Th1/Th2 Response may be Associated with Higher Risk of Relapse

**Fig. 3:**
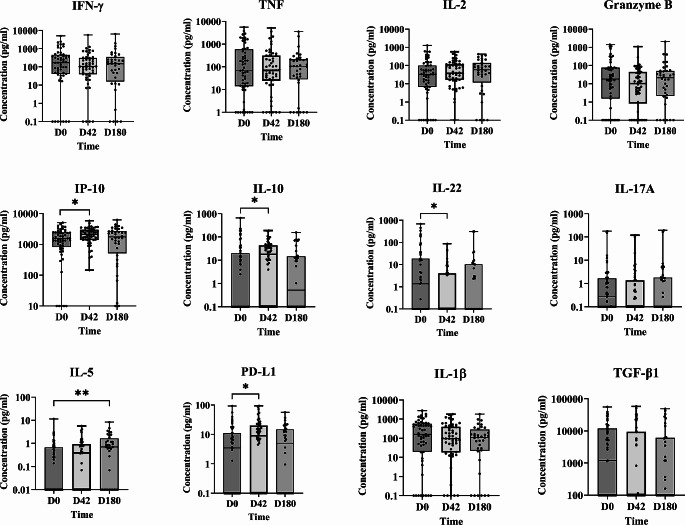
Cytokine, PD-L1 and granzyme B concentrations in plasma from patients treated with LAmB/MF (Arm 2) (as determined by WBA) in the mITT set.  IFN-γ, TNF, IL-2, granzyme B, IP-10, IL-10, IL-22, IL-17A, IL-5, PD-L1, IL-1β and TGF-β1 concentrations (pg/ml) were measured at D0 (55 patients), D42 (50 patients) and D180 (36 patients). Statistical differences (Mann-Whitney test) *p<0.05, **p<0.01

The patients who received LAmB/MF treatment also showed high concentrations of proinflammatory cytokine at D0, and low concentrations of IL-5, IL-13, and IL-10 (Fig. 3). However, unlike in Arm 1, no significant changes were observed in most of the Th1 cytokines studied over follow-up (Fig. 3). Rather, a significant increase in IP-10, IL-10 and PD-L1 was detected [(median (IQR; *p value*) D0 vs. D42 = 1535 (0-5041) vs. 2108 (146.20–5663; <0.05); 0 (0-659.30) vs. 17.91 (0-189; <0.05); 3.46 (0-93.32) vs. 8.98 (0-94.50; <0.05), respectively)], along with a reduction in IL-22 at D42 [(median (IQR; *p value*) = 1.35 (0-683.70) at D0 vs. 0 (0-86.73; *p* = 0.017) at D42], and an increase in IL-5 at D180 [(median (IQR; *p value*) = 0 (0-11.45) at D0 vs. 0.70 (0-8.36; *p* = 0.0021) at D180] (Fig. 3), all indicative of a Th1/Th2 response. At 12 months, the cure rate recorded with LAmB/MF was 80% in the mITT set and 86.3% in the set of completers, with 1 patient not responding to therapy, another one with an AE of hypersensitivity related to LAmb who received rescue treatment after treatment discontinuation, and 5 of the 55 patients relapsing between D180 and 12 months.

### Relapsed Patients Showed a Depleted Specific Cellular Response at D0

**Fig. 4:**
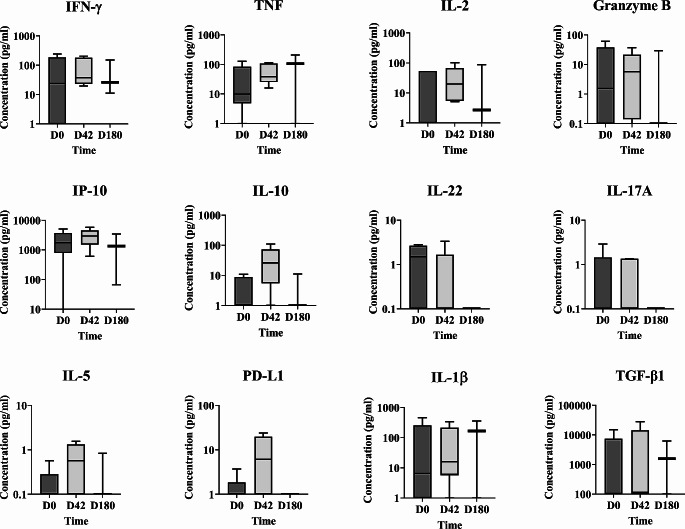
Cytokine, PD-L1 and granzyme B concentrations in plasma from patients who relapsed (as determined by WBA) in the mITT set. IFN-γ, TNF, IL-2, granzyme B, IP-10, IL-10, IL-22, IL-17A, IL-5, PD-L1, IL-1β and TGF-β1 concentrations (pg/ml) were measured at D0 (5 patients), D42 (5 patients) and D180 (3 patients).

**Fig. 5:**
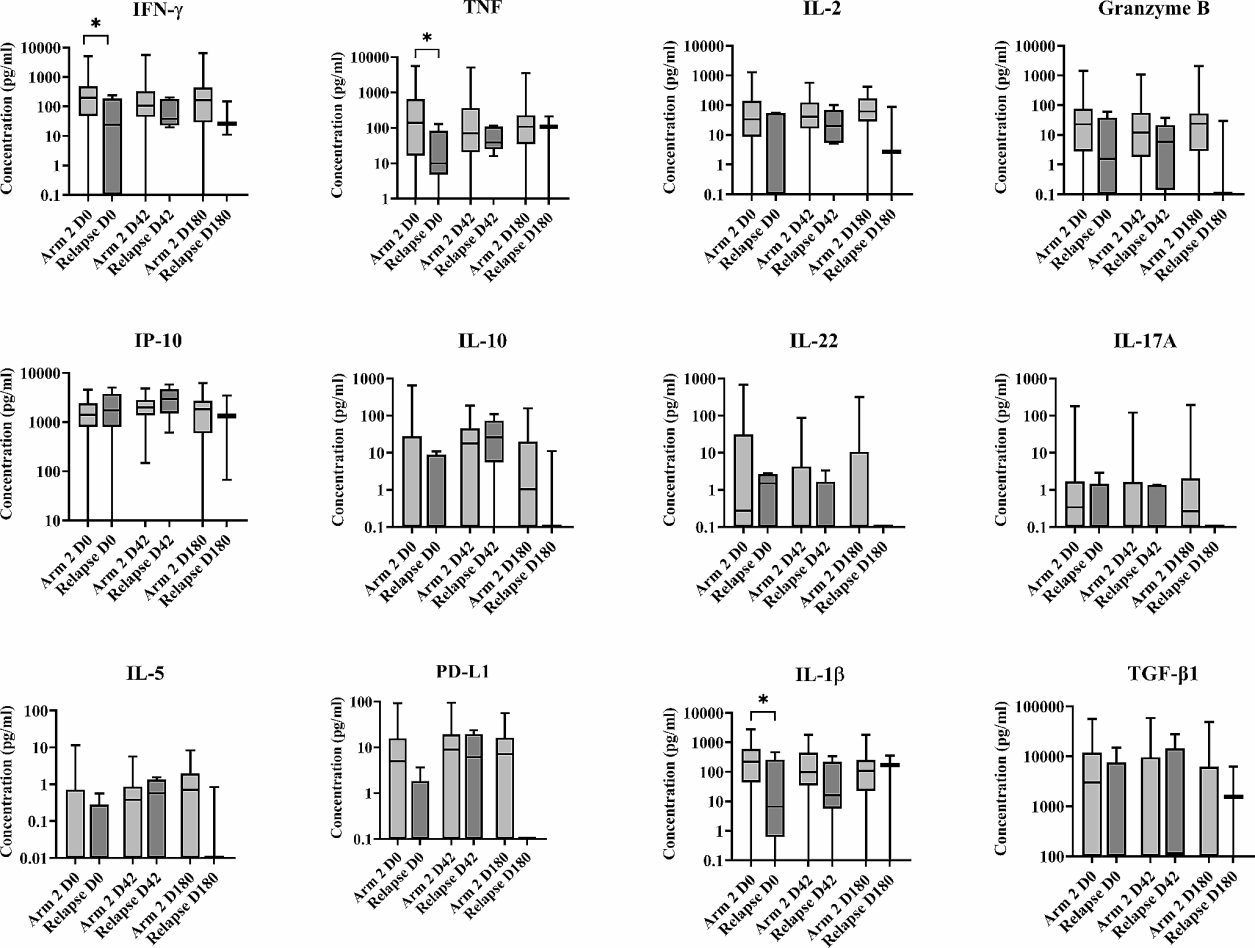
Comparison of cytokine, PD-L1 and granzyme B concentrations in plasma from all Arm 2 patients (n= 50) and relapsed patients (only seen in Arm 2) (as determined by WBA) in the mITT set. IFN-γ, TNF, IL-2, granzyme B, IP-10, IL-10, IL-22, IL-17A, IL-5, PD-L1, IL-1β and TGF-β1 concentrations (pg/ml) were measured at D0 (Arm 2 = 50 patients; Relapsed= 5 patients), D42 (Arm 2 = 45 patients; Relapsed = 5 patients), and D180 (Arm 2 = 33 patients; Relapsed = 3 patients). Statistical differences (Mann-Whitney test) *p<0.05

Given that the LAmB/MF treatment cured 80% of the patients who received it (86.3% in the complete case scenario), indicating it to be effective in most people, analyses were made to determine the profile of patients with higher risk of failure. All patients who clinically relapsed showed a depleted immune cell response to *Leishmania* at D0 (Fig. 4, Table S1). While these patients did show clinical improvement after LAmB/MF treatment, this was not sustained over time, unlike those who were eventually cured in the same Arm (Fig. 5). The same pattern was recorded when all patients from both arms were included in the analysis (Fig. S2).

Differences in the IFN-γ and IL-10 profiles for control and SLA-stimulated plasma during follow-up were also examined (Table 1). In the controls, IL-10 was detected at D0, but this decreased by D42 in the Arm 2 patients (*p* = 0.0264), returning to around D0 concentrations by D180. For both treatments, higher concentrations of IFN-γ than IL-10 were detected in SLA-stimulated plasma, except in the case of relapsed patients, who showed the opposite [IFN-γ/IL-10: Arm 1 73.08/48.52, Arm 2 108.40/55.33, Relapsed: 40.21/75.15 at D42; Arm 1 200.60/95.52, Arm 2 161.80/73.91, Relapsed: 26.02/132.70 at D180] (Table 1).

**Table 1:** Concentrations (median values) of IFN-γ and IL-10 in control plasma and SLA-stimulated plasma before and after treatment (Arm 1 and Arm 2 patients, and relapsed patients [seen in Arm 2 only])

	D0				D42				D180			
	IFN-γ		IL-10		IFN-γ		IL-10		IFN-γ		IL-10	
	Control	SLA	Control	SLA	Control	SLA	Control	SLA	Control	SLA	Control	SLA
ARM 1	0	221.8a*	56.03	75.38	0	73.08b*	37.96	48.52	0	200.6	60.03	95.52
ARM 2	3.952	177.1	59.37 A*	48.45	1.022	108.4	28.67B*	55.33	0	161.8	42.59	73.91
RELAPSED	5.379	31.92	29.81	11.94	2.044	40.21	36.3	75.15	0	26.02	139.5	132.7

Data are presented as the concentration (pg/mL) of the different cytokines (IFN-γ and IL-10) and statistical differences (Mann-Whitney test (*p < 0.05)) are presented as follows:

Controls A: D0-D42, B: D42-D180, C: D0-D180. SLA a: D0-D42, b: D42-D180, c: D0-D180

Sample sizes: D0 (Arm 1 and Arm 2: 55 patients; Relapsed: 5 patients), D42 (Arm 1: 54 patients; Arm 2: 50 patients; Relapsed: 5patients), D180 (Arm 1 and Arm 2: 36 patients; Relapsed: 3 patients)

Finally, several haematological and biochemical parameters were evaluated comparing relapsed patients with those who did not relapse (Table S2). It revealed that no substantial differences existed beforehand between relapsed and non-relapsed patients that could explain the poor Th1 response or the cause of relapse.

### In Arm 2, Low IFN-γ Producers at D0 Produced Low IFN-γ and High IL-10 Concentrations after Treatment

**Fig. 6:**
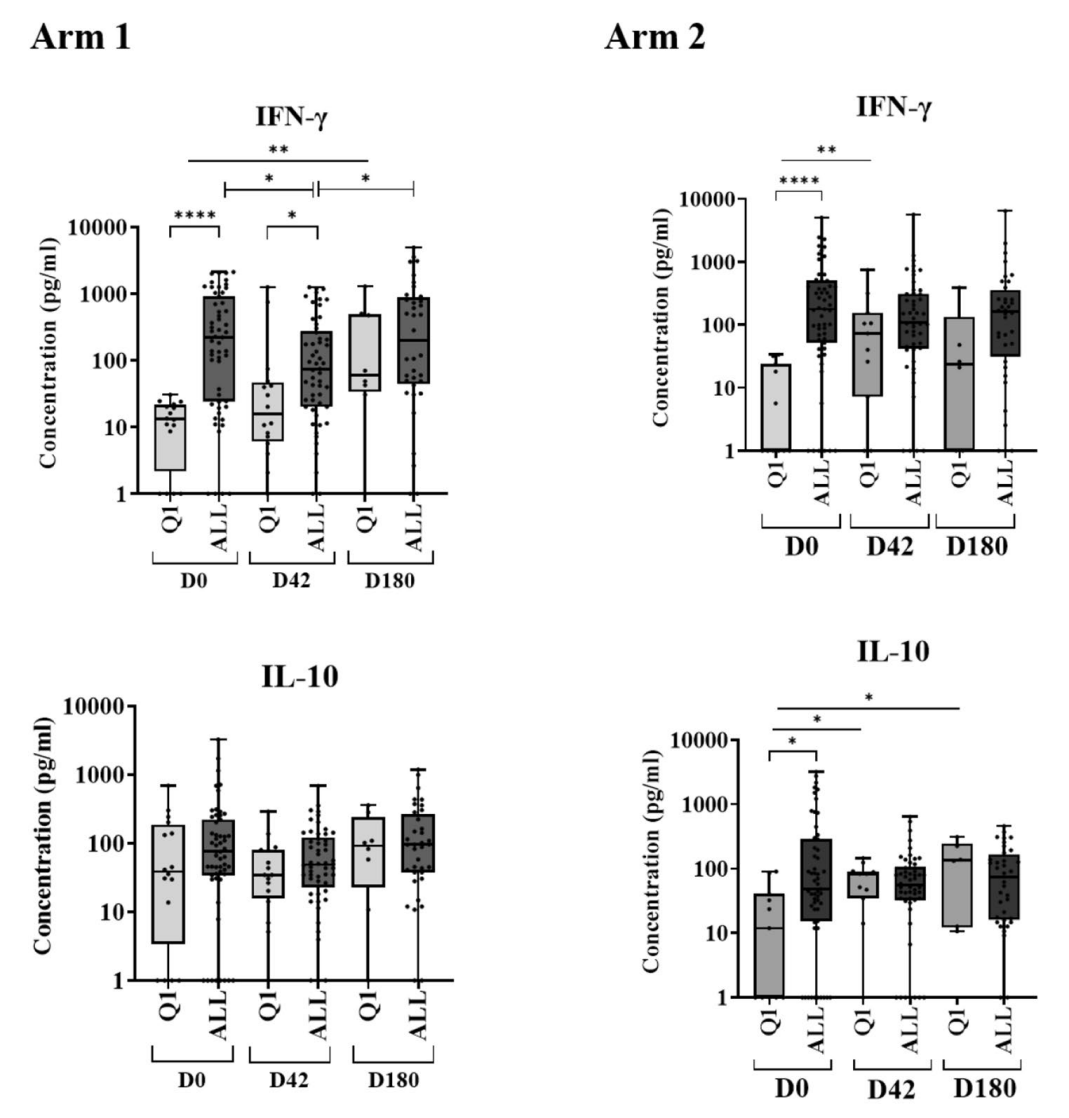
IFN-γ and IL-10 concentrations in plasma from low IFN-γ producers (Q1) and all patients (as determined by WBA) in the mITT set. IFN-γ and IL-10 concentrations (pg/ml) were measured at screening (D0), end of treatment (D42) and 6 months of follow-up (D180). Statistical differences (Mann-Whitney test) are expressed as *p < 0.05; **p < 0.01; ****p < 0.0001

To determine whether any non-relapsing patients (from Arm 1 or Arm 2) shared similar characteristics as those of the relapsed Arm 2 patients, the interquartile ranges (IQR) for IFN-γ production by all patients at D0 were calculated, and low IFN-γ producers were identified (quartile 1 [Q1]) (Fig. 6). Among the Arm 1 low IFN-γ producers (arm where all participants were considered cured at 12 months of follow-up), higher IFN-γ concentrations were seen at D180 than at D0 [(median (IQR; *p value*) D0 = 13.18 (0-30.38) vs. D180 = 59.41 (0-1281); *p* = 0.0012)], with no changes in IL-10 (Fig. 6). Among the Arm 2 low IFN-γ producers, higher IFN-γ concentrations were seen at D42 than at D0 [(median (IQR) D0 = 0 (0-33.68); D42 = 73.08 (0-743.30; *p value* = 0.0083), respectively)], with no further increase by D180. It is important to highlight that IL-10 was increased in this group at D42 and at D180 [(median (IQR; *p value*) D0 = 11.98 (0-90.53); D42 = 83.21 (0-146.60; *p* < 0.05); D180 = 135.60 (10.71–310.30; *p* < 0.05), respectively)] (Fig. 6).

## Discussion

At D0, the SLA-stimulated plasma of most of the present 110 patients revealed a mixed cellular response characterized by high median concentrations of TNF, granzyme B, IFN-γ, and TGF-β1, and low concentrations of IL-5, IL-13 and IL-10. This complements the results of an earlier study that revealed high IFN-γ and low IL-10 concentrations after SLA stimulation of PBMCs from Sudanese patients with PKDL [12, 13]. Both sets of results indicate the presence of circulating *Leishmania*-specific Th1 cells in most patients before treatment (similar to that seen in patients with cutaneous leishmaniasis or cured VL [18, 19]) but also of Th2 cells. In this context, the accumulation of M2 macrophages would lead to increased concentrations of TNF and TGF-β (among other cytokines), exacerbating the ongoing inflammatory immune response [20, 21]. Thus, the systemic Th1/Th2 immune response developed after therapy for VL in the present patients, while sufficient to maintain low parasite loads, was unable to prevent the development of PKDL in some individuals [22]. A possible explanation for this may lie in the fact that most of the present patients were children (the population most affected by PKDL in Sudan [5, 22]), and children have lower frequencies of Th1, Th2 and Th17 cells, and higher numbers of resting T regulatory (Treg) cells compared with adults [23, 24]. To prevent PKDL from appearing, attempts to induce a stronger Th1 response using safe immunomodulatory agents alongside VL treatment have been made [14, 25], but the strategy needs further development.

At the end of PM/MF treatment (D42, Arm 1), a reduction was seen in the production of TNF. In the LAmB/MF-treated patients (Arm 2), an increase in IP-10, IL-10 and PD-L1 was seen, along with a reduction in IL-22. The latter treatment, which was shorter but less effective (80% cure rate) [15] was unable to induce a Th17 response, and clearly increased the expression of the regulatory cytokine IL-10 and of the exhaustion marker PD-L1. The absence of Th17 cytokines (such as IL-17A, IL-17F and IL-22) has been associated with the pathogenesis of PKDL [20, 26]. In addition, the concomitant upregulation of PD-1 and IL-10 might indicate the existence of anergic/exhausted CD8 + T cells, which would be poorly responsive [27].

At D180, the PM/MF-treated patients showed increased concentrations of granzyme B, IFN-γ, IL-2, IL-1β and IL-17A compared to D42, while no changes were detected in the LAmB/MF-treated patients. The induction of these Th1 and Th17 responses seemed to be more effective in the clinical resolution of PKDL.

At D180, no meaningful changes were seen in the cytokine load compared to D0, except for a significant increase in IL-17A and IL-10 in the PM/MF-treated patients, and in IL-5 in those treated with LAmB/MF. IL-17A is an inducer of TNF, IFN-γ and nitric oxide levels in both PKDL lesions and in the blood, while IL-5 is a Th2 cytokine [28–30].

The stimulation of blood cells with SLA resulted in reduced TNF production in thePM/MF-treated patients, perhaps caused by the presence of IL-10. In these patients, the production of Th1 cytokines in the absence of high concentrations of Th2 cytokines or TGF-β1, suggests that the cells producing IL-10 might be Th1 cells or T regulatory (Treg) cells [31, 32]. Difficulties in obtaining PBMCs at the clinical trial site meant that no additional CD4 + or CD8 + T cell-derived Th1 or Th2-related cytokines could be sought to confirm this hypothesis.

A stronger Th1 response to treatment might have been expected given the cytokine/other analyte profiles of the patients at D0. However, unlike for other clinical manifestations of leishmaniasis, the literature contains no evidence that a systemic Th1 response occurs in PKDL. In fact, the only two studies describing the specific systemic immune response before and after PKDL treatment in Sudanese patients showed no significant differences in IFN-γ and IL-4 or IL-10 concentrations [14, 33].

At 12 months, five patients who received LAmB/MF required rescue treatment because they relapsed. Analysis of the cytokine profile of this sub-group at D0 showed that IFN-γ, TNF and IL-1β concentrations were significantly lower than in patients in Arm 2 who were eventually cured. Until D180, the patients who relapsed had clinical improvement, but their *Leishmania*-specific lymphocyte reactivity was incapable of fully resolving the disease, and PKDL lesions reappeared. No obvious reason for this depleted immune response could be identified. In the clinical trial, the study population homogeneity reduced the risk of bias being introduced by factors like lesion type, lesion grade and VL treatment on the PKDL treatment outcome. In addition, comparison of the haematological and biochemical parameters of the relapsed patients with those who did not relapse resulted in similar profiles, which suggests that the differences observed in the cytokine profile of those patients may be attributed to the action of the treatment and the underlying mechanisms to overcome the disease. Considering this, patients with a poor specific Th1 response at D0 may be more likely to relapse if treated with LAmB/MF. Screening before treatment could therefore guide treatment recommendation. This is further supported by the response seen in the low IFN-γ producers at D0. When treated with PM/MF, these patients showed higher IFN-γ concentrations at D180 than at D0, with no changes in IL-10. However, low IFN-γ producers who were treated with LAmB/MF exhibited no change in IFN-γ concentrations compared to D0, and IL-10 was increased at D42 and D180, and it was only with this treatment that relapses were observed. Clearly, different immune responses were induced by the two treatments in low IFN-γ producers. It seems that higher IFN-γ concentrations and the absence of IL-10 could be associated with a more effective treatment against PKDL.

Although some biomarkers of disease progression, such as IFN-γ, TNF, IL-10, TGF-β and PD-1, have been described in the skin of Sudanese PKDL patients [20], until now no blood biomarker was associated with an adequate response to PKDL treatment or drug combination. Measuring IFN-γ, TNF and IL-10 via the WBA could provide a useful tool to monitor patients, detecting those at higher risk of poor response to treatment, and to evaluate new interventions. However, this should be confirmed with a larger number of patients.

Despite the above results, measuring the magnitude of the systemic cellular response via the production of Th1, Th2 or Th17 cytokines may not reflect the immune system full functional potential. The acquisition of systemic protective immunity may not reflect a skin-specific immune response [34]. More studies regarding the magnitude and quality of the cellular response are needed to shed light on this.

In conclusion, this study partially reveals the specific cellular immune response that occurs in patients with PKDL during and after treatment with two new drug combinations - an important milestone in the evaluation of novel treatments for PKDL that highlights the potential of the WBA in this context. Both treatments were effective in treating patients with PKDL. However, low IFN-γ producers at treatment onset had higher risk of failure when treated with LAmB/MF, explaining partially the differences in treatment outcome between the two treatment options. Screening for low IFN-γ could support treatment recommendation and should be included in future studies of PKDL therapy. While the positive results of this trial represent a significant improvement over existing options, the elimination of VL from East Africa will ultimately require easier-to-administer oral therapies that can be given to all patients with PKDL. The usefulness of such treatments could be investigated with the technique used in the present work.

## Electronic Supplementary Material

Below is the link to the electronic supplementary material.

Supplementary Material 1

Supplementary Material 2

Supplementary Material 3

## Data Availability

Data is provided within the manuscript or supplementary information files.

## Abbreviations

IFN: Interferon
IL: Interleukin
LAmB: Liposomal amphotericin B [AmBisome^®^]
MF: Miltefosine
PBMCs: Peripheral blood mononuclear cells
PHA: Phytohemagglutinin
PKDL: Post-kala-azar dermal leishmaniasis
PM: Paromomycin
SLA: Leishmania soluble antigen
SSG: Sodium stibogluconate
TGF: Transforming growth factor
Th: T helper
TNF: Tumor necrosis factor
VL: Visceral leishmaniasis
WBA: Whole blood assay
