# Supplementary material for: Differences in the Cellular Immune Response during and after Treatment of Sudanese Patients with Post-kala-azar Dermal Leishmaniasis, and Possible Implications for Outcome
Source: J Epidemiol Glob Health. 2024 Jul 15;14(3):1167–79. doi: 10.1007/s44197-024-00270-0 (PMC11442715; doi:10.1007/s44197-024-00270-0)

**S1 Figure. Cytokine pattern of all patients regarding the grade of PKDL lesions at screening.** IFN-γ, TNF, IL-2, granzyme B, IL-5, IL-10, IL-22, IL-17A and TGF-β1 concentrations (pg/ml) were measured at screening (D0; Grade 1 = 67 patients vs Grade 2&3 = 43 patients), end of treatment (D42; Grade 1 = 61 patients vs Grade 2&3 = 42 patients) and 6 months of follow-up (D180; Grade 1 = 51 patients vs Grade 2&3 = 30 patients).

**
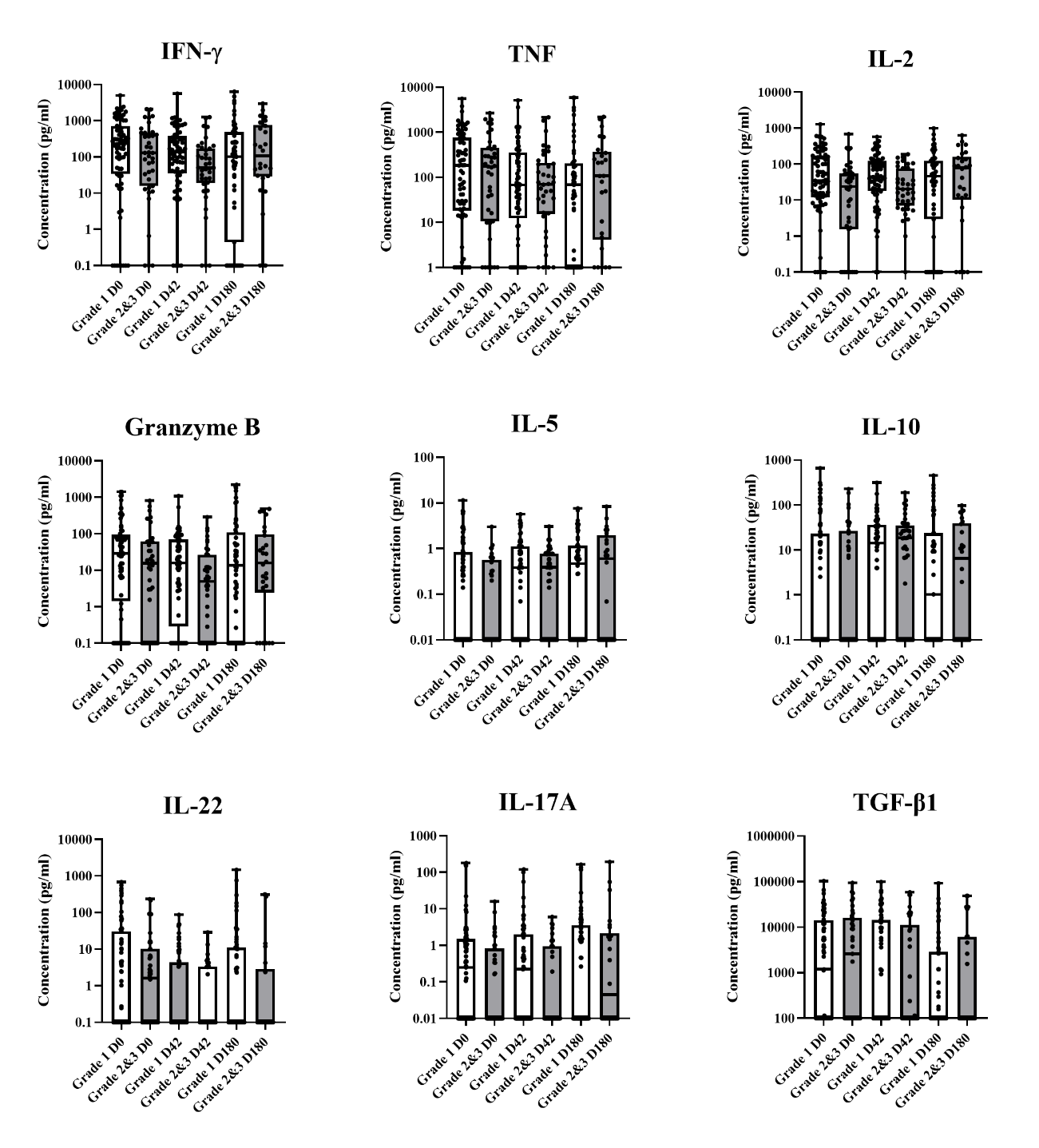
**

**S2 Figure. Comparison of cytokine, PD-L1 and granzyme B production in whole blood plasma between all cured patients (arm 1 and arm 2) and relapsed patients after 24h of SLA stimulation.** IFN-γ, TNF, IL-2, granzyme B, IP-10, IL-10, IL-22, IL-17A IL-5, PD-L1, IL-1β and TGF-β1 concentrations (pg/ml) were measured at screening (D0; All = 105 patients vs Relapsed = 5 patients), end of treatment (D42; All = 99 patients vs Relapsed = 5 patients) and 6 months of follow-up (D180; All = 69 patients vs Relapsed = 3 patients). Statistical differences (Mann-Whitney test) *p<0.05


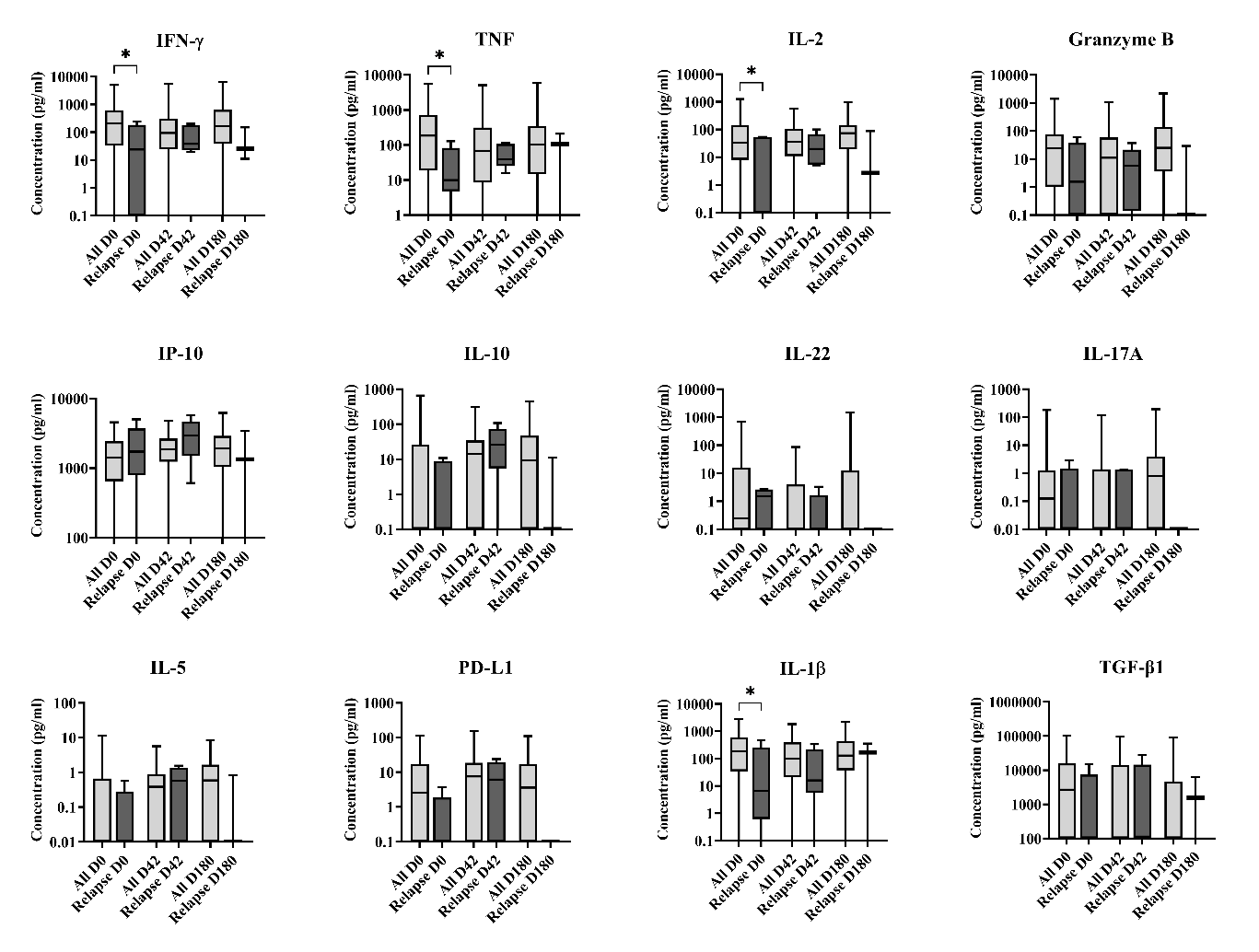

Supplement: Supplementary file 1 — Supplementary Material 1 [file 44197_2024_270_MOESM1_ESM.docx]
