# Supplementary material for: Differences in the Cellular Immune Response during and after Treatment of Sudanese Patients with Post-kala-azar Dermal Leishmaniasis, and Possible Implications for Outcome
Source: J Epidemiol Glob Health. 2024 Jul 15;14(3):1167–79. doi: 10.1007/s44197-024-00270-0 (PMC11442715; doi:10.1007/s44197-024-00270-0)
Supplement: Supplementary file 2 — Supplementary Material 2 [file 44197_2024_270_MOESM2_ESM.docx]

**Supporting information**

**S1 Table. Evaluation of the 15 analytes measured in SLA-stimulated plasma (median (pg/mL); (IQR)).**

|  | **TOTAL** | | | **ARM 1** | | | **ARM 2** | | | **RELAPSED PATIENTS** | | |
| --- | --- | --- | --- | --- | --- | --- | --- | --- | --- | --- | --- | --- |
|  | **D0** | **D42** | **D180** | **D0** | **D42** | **D180** | **D0** | **D42** | **D180** | **D0** | **D42** | **D180** |
|  | **Median (IQR)** | **Median (IQR)** | **Median (IQR)** | **Median (IQR)** | **Median (IQR)** | **Median (IQR)** | **Median (IQR)** | **Median (IQR)** | **Median (IQR)** | **Median (IQR)** | **Median (IQR)** | **Median (IQR)** |
| **IFN-γ** | 187.80  (0-5038) | 94.13  (0-5639) | 148.10  (0-6430) | 212.70  (0-2113) | 69.85  (0-1244) | 190.90  (0-4726) | 171.60  (0-5038) | 107  (0-5639) | 148.10  (0-6430) | 24.06  (0-242.50) | 38.17  (19.40-203.20) | 26.02  (11.13-150.20) |
| **TNF** | 175.60  (0-5573) | 68.15  (0-5093) | 104.40  (0-5844) | 244.20  (0-1612) | 57.63  (0-1342) | 90.15  (0-5844) | 71.27  (0-5573) | 70.16  (0-5093) | 106.80  (0-3523) | 9.85  (0-128.3) | 38.70  (15.92-114.10) | 106.90  (0-210.30) |
| **IL-2** | 33.19  (0-1277) | 34.26  (0-569.7) | 67.86  (0-989.1) | 33.56  (0-679.40) | 27.34  (0-377.70) | 91.67  (0-989.10) | 33.05  (0-1277) | 38.32  (0-569.70) | 57.81  (0-420.70) | 0  (0-54.32) | 20.11  (5.15-101.60) | 2.70  (0-88.83) |
| **Granzyme B** | 22.37  (0-1409) | 10.14  (0-1088) | 24.47  (0-2210) | 24.43  (0-1407) | 10.14  (0-540.10) | 25.93  (0-2210) | 17.93  (0-1409) | 10.19  (0-1088) | 21.21  (0-2035) | 1.55  (0-60.28) | 5.78  (0-37.13) | 0  (0-29.10) |
| **IP-10** | 1506  (0-5041) | 1941  (0-5809) | 1888  (0-6209) | 1478  (0-4334) | 1780  (0-4037) | 1923  (9.66-4920) | 1535  (0-5041) | 2108  (146.20-5809) | 1736  (0-6209) | 1737  (0-5041) | 2953  (611.30-5809) | 1341  (66.72-3449) |
| **IL-10** | 0  (0-659.30) | 15.71  (0-311.50) | 9.18  (0-453.50) | 0  (0-313.60) | 13.66  (0-311.50) | 11.34  (0-453.50) | 0  (0-659.30) | 17.91  (0-189) | 0.51  (0-157.70) | 0  (0-10.84) | 26.10  (0-108.80) | 0  (0-11.13) |
| **IL-22** | 0.26  (0-683.70) | 0  (0-86.73) | 0  (0-1473) | 0  (0-566.20) | 0  (0-48.12) | 0  (0-1473) | 1.35  (0-683.70) | 0  (0-86.73) | 0  (0-313.10) | 1.49  (0-2.79) | 0  (0-3.30) | 0  (0-0) |
| **IL-17A** | 0.05  (0-180.50) | 0  (0-120.10) | 0.46  (0-192) | 0  (0-22.27) | 0  (0-55.25) | 1.29  (0-137) | 0.27  (0-180.50) | 0  (0-120.10) | 0  (0-192) | 0  (0-2.88) | 0  (0-1.36) | 0  (0-0) |
| **IL-5** | 0  (0-11.45) | 0.39  (0-5.71) | 0.59  (0-8.36) | 0.19  (0-6.75) | 0.39  (0-3.51) | 0.53  (0-7.60) | 0  (0-11.45) | 0.39  (0-5.71) | 0.70  (0-8.36) | 0  (0-0.57) | 0.57  (0-1.56) | 0  (0-0.84) |
| **PDL-1** | 1.80  (0-113.80) | 7.32  (0-154.70) | 2.19  (0-109.10) | 0  (0-113.80) | 4.32  (0-154.70) | 1.49  (0-109.10) | 3.46  (0-93.32) | 8.98  (0-94.50) | 4.81  (0-56.69) | 0  (0-3.70) | 6.14  (0-23.96) | 0  (0-0) |
| **IL-1β** | 166.30  (0-2772) | 96.03  (0-1814) | 131.10  (0-2193) | 168.10  (0-2318) | 84.48  (0-1391) | 204  (0-2193) | 164.40  (0-2772) | 96.03  (0-1814) | 110.30  (0-1817) | 6.63  (0-463.30) | 16.02  (0-337.80) | 167.90  (0-356.70) |
| **TGF-β1** | 2011  (0-103,847) | 0  (0-98,858) | 0  (0-92,407) | 2271  (0-103,847) | 119.40  (0-98,858) | 0  (0-92,407) | 1188  (0-55,943) | 0  (0-58,522) | 79.81  (0-48,727) | 0  (0-15,026) | 114.30  (0-28,119) | 1566  (0-6171) |
| **IL-4** | 0  (0-5.17) | 0  (0-15.18) | 0  (0-43.53) | 0  (0-3.20) | 0  (0-2.67) | 0  (0-43.53) | 0  (0-5.17) | 0  (0-15.18) | 0  (0-0) | 0  (0-0) | 0  (0-0) | 0  (0-0) |
| **IL-13** | 0.64  (0-109.10) | 2.07  (0-101.20) | 2.84  (0-105.10) | 0  (0-29.64) | 0.87  (0-101.20) | 3.08  (0-33.93) | 0.87  (0-109.10) | 4.57  (0-97.14) | 2.83  (0-105.10) | 3.47  (0-13.78) | 9.82  (0-39.73) | 4.33  (0-4.69) |
| **IL-23** | 0  (0-4.80) | 0  (0-5.83) | 0  (0-10.95) | 0  (0-4.80) | 0  (0-5.83) | 0  (0-2.46) | 0  (0-2.46) | 0  (0-3.95) | 0  (0-10.95) | 0  (0-0) | 0  (0-3.95) | 0  (0-8.33) |

Data reported is the difference between SLA and control concentrations. Statistical differences are not shown in this table
