# Supplementary material for: Differences in the Cellular Immune Response during and after Treatment of Sudanese Patients with Post-kala-azar Dermal Leishmaniasis, and Possible Implications for Outcome
Source: J Epidemiol Glob Health. 2024 Jul 15;14(3):1167–79. doi: 10.1007/s44197-024-00270-0 (PMC11442715; doi:10.1007/s44197-024-00270-0)
Supplement: Supplementary file 3 — Supplementary Material 3 [file 44197_2024_270_MOESM3_ESM.docx]

**Supporting information**

**S2 Table. Evaluation of the haematological and biochemical parameters measured in the relapsed patients and the rest of the mITT set at baseline.**

| **Parameter** | **Relapsed patients** | **Non-relapsed patients** | ***p*-value relapsed vs non-relapsed** |
| --- | --- | --- | --- |
| **Albumin g/L**  **(Low:30;Upper:50)** | 39.30 ± 5.86 | 39.13 ± 4.27 | 0.77 |
| **Creatinine mg/dL**  **(Low:0.2;Upper:1.4)** | 0.4 ± 0.14 | 0.41 ± 0.20 | 0.94 |
| **Pottasium mmol/L**  **(Low:3;Upper:5)** | 4.14 ± 0.32 | 3.97 ± 0.33 | 0.28 |
| **SGOT/AST U/L**  **(Upper:<=40)** | 35 ± 4.06 | 30.91 ± 10.18 | 0.12 |
| **SGPT/ALT U/L**  **(Upper:<=40)** | 25.8 ± 9.44 | 26.57 ± 12.53 | 0.99 |
| **Total Bilirubin mg/dL**  **(Upper:<=1.2)** | 0.40 ± 0.24 | 0.48 ± 0.29 | 0.61 |
| **Basophils %** | 0 ± 0 | 0 ± 0 | ND |
| **Eosinophils %** | 2.60 ± 2.51 | 2.41 ± 1.56 | 0.69 |
| **Haematocrit %** | 36 ± 2.24 | 36.94 ± 3.57 | 0.47 |
| **Haemoglobin g/dL**  **(Low:12;Upper:17.5)** | 11.56 ± 1.20 | 12.45 ± 1.26 | 0.13 |
| **Lymphocytes %** | 45 ± 14.40 | 45.58 ± 8.16 | 0.88 |
| **Monocytes %** | 7 ± 3.24 | 7.87 ± 2.83 | 0.68 |
| **Neutrophil %** | 45.20 ± 9.58 | 44.15 ± 8.92 | 0.79 |
| **Platelets x103/µL**  **(Low:150;Upper:500)** | 345 ± 111.88 | 341.40 ± 95.17 | 0.60 |
| **RBC x 10^6^/µL** | 4.74 ± 0.38 | 4.68 ± 0.47 | 0.61 |
| **WBC x 10^6^/µL**  **(Low:4; Upper:10)** | 7.46 ± 1.07 | 7.14 ± 3.47 | 0.30 |

Data reported is presented as concentrations or percentages of each parameter. Statistical differences were evaluated by Mann-Whitney test.
